# Development of a Secondary Use Method for Non-Ferrous Slags Metallurgy for Obtaining Mineral Fertilizers

**DOI:** 10.3390/ijms27104470

**Published:** 2026-05-16

**Authors:** Alfira Sabitova, Rystay Mukiyanova, Zhanar Kassymova, Bulbul Bayakhmetova

**Affiliations:** Department of Chemistry and Ecology, Shakarim University, Semey 071400, Kazakhstan; alfa-1983@mail.ru (A.S.); kasymova-z@mail.ru (Z.K.); bulbul.bayakhmetova@mail.ru (B.B.)

**Keywords:** metallurgical slag, mineral fertilizer, chemical leaching, waste utilization, corn

## Abstract

This study explores the use of metallurgical slag extracts as a liquid mineral fertilizer for maize cultivation. Slag samples were obtained from the former lead smelter in Shymkent and the Zhezkent Mining and Processing Plant. Elemental analysis identified the slag from the second storage area of the Shymkent smelter as the least contaminated with potentially toxic elements and enriched in macro- and micronutrients. Slag extraction was conducted via chemical leaching using potassium sulfate and ammonia solutions in a hydrogen peroxide medium, yielding Cu^2+^ and Zn^2+^ concentrations of 423.751 mg/L and 86.649 mg/L, respectively. The resulting extracts were diluted with distilled water at a ratio of 1:10 (potassium sulfate extract) and 1:200 (ammonia extract) and applied to assess early seed development and subsequent maize yield. Seed germination rates were comparable to those of the control group (100%). After 90 days of growth, maize plants treated with the ammonia-based extract showed positive effects on root system development, stem growth, and cob formation. The concentration of potentially toxic elements in the dry plant biomass remained within permissible limits. These findings demonstrate the potential for the safe agricultural use of these extracts while ensuring the rational utilization of industrial waste.

## 1. Introduction

The processing of metal-containing mineral waste [1] remains a global challenge, as the metallurgical industry is a primary source of environmental contamination by highly toxic metals (Pb, As, Cd) and hazardous gases such as H_2_S and SO_2_ [2]. Large volumes of solid waste are generated at both former and operating metallurgical enterprises, with slags forming extensive waste heaps [3]. One of the largest slag dumps in Europe is located in Poland; its volume, even with regular processing, exceeds 600 million tons [4]. In Kazakhstan, the total volume of waste from non-ferrous metallurgy exceeds 5 billion tons, occupying more than 13,000 hectares of land [5]. The volume of accumulated metallurgical waste in the tailing dump of the Zhezkent Mining and Processing Plant (MPP) is over 1 billion tons [4], while the former lead smelter in Shymkent contains approximately 1.9 million tons [6,7].

The quantity of slag generated depends on the amount of ferrous or non-ferrous metal produced. Non-ferrous metallurgy is associated with high levels of slag formation [8,9]. Table 1 presents the volumes of slag generated per ton of metal produced.

The main chemical components of slags (wt.%) from Zn and Pb production are as follows: FeO_total_ 0.88–59.6; SiO_2_ 2.04–57.1; CaO 0.18–32.23; MgO 0.61–15.9; ZnO 0.03–47.3; PbO 0.002–6.4 [20,21]. Table 2 shows the concentrations of chemical elements that are contained in metallurgical slags that are potentially toxic to soils and plants [3].

According to Table 2, non-ferrous metallurgy slags may have a significantly higher environmental impact than ferrous metallurgy slags. To mitigate these impacts and ensure waste-free production, slags can be used as a secondary raw material source for metal recovery through hydrometallurgical methods and bioleaching [20,24]. The hydrometallurgical method is based on leaching slags with solutions of acids, salts, or alkalis, resulting in the transfer of metals into solution in the form of Me^n+^ ions (Reactions 1, 2):

ZnFe + 4H_2_SO_4_ = ZnSO_4_ + Fe_2_(SO_4_)_3_ + 4H_2_O (1)

Ca[Zn(OH)_3_]_2_ · H_2_O + 3H_2_SO_4_ = CaSO_4_ + 2ZnSO_4_ + 8H_2_O (2)

During bioleaching, metals are transferred into solution by microorganisms under acidic conditions at pH 1.5–3.0 (Reactions 3, 4) [25]:

4FeS_2_ + 14O_2_ + 4H_2_O → 4FeSO_4_ + 4H_2_SO_4_ (3)

4FeSO_4_ + O_2_ + 2H_2_SO_4_ → 2Fe_2_(SO_4_)_3_ + 2H_2_O (4)

In both approaches, metals are subsequently extracted from solution by solvent extraction, electrolysis or precipitation.

In addition, metallurgical slags are utilized as fillers in construction materials (e.g., road engineering, concrete, and cement) due to their favorable physical and mechanical properties, such as density, hardness, and melting point [20,26].

Furthermore, metallurgical slags offer a practical and cost-effective solution for wastewater treatment. They have been used to remediate acid mine drainage in the Witwatersrand Basin and the Mpumalanga coalfields in South Africa and serve as a substrate in vertical flow-through wetlands for municipal wastewater treatment in La Motte d’Aigues, France [27].

Current research of the beneficial effects of the physicochemical modification of various slag types on crop yields and soil fertility is summarized in Table 3.

Based on the findings presented in Table 3, it can be concluded that metallurgical slags possess several agronomically valuable properties. These benefits include a diverse array of macro- and microelements, improved soil structure and moisture retention, enhanced plant growth, and increased stress resistance. Macro- and microelements such as Ca, Mg, Cu, Fe, Mn, Si, and Zn are of key importance for plant growth and development [37,38,39]. Further study of the possibility of using slag in agriculture contributes to environmentally friendly disposal, reduces industrial waste, and increase in soil fertility and the sustainability of crop production. Currently, two main methods are used for applying metallurgical slags as fertilizer: application in solution form or as liquid/solid formulations. When processing slags into liquid fertilizers, metals are converted into soluble forms, which are more easily absorbed by plants [20].

This study evaluates a method for obtaining liquid fertilizers from metallurgical slags via chemical leaching (extraction). The biological activity of the resulting extracts was assessed through bioassays conducted on various organs of the maize plant (*Zea mays* L.). This high-biomass species is widely used in ecotoxicological research because of its sensitivity to chemical pollutants and its capacity to accumulate heavy metals [40,41,42,43]. Bioassays of the developed fertilizer allowed evaluation of the bioavailability of metal compounds and their uptake by *Zea mays* L. plants.

## 2. Results and Discussion

### Characteristics of the Granulometric Composition of Metallurgical Slags

According to literature data [44,45,46], slag particle size significantly affects leaching efficiency, as it influences both the specific surface area and the distribution of mineral phases. For instance, reducing the particle size to d_80_ < 10 µm increases Cu^2+^ recovery by 7% during flotation [44]. A similar trend was observed for electric arc furnace slags [45], where increasing the particle size within the 4–10 mm range led to decreased concentrations of Ba^2+^, Ca^2+^, and Sr^2+^ in aqueous extracts. Furthermore, a clear correlation exists between the particle size of steelmaking slags and their chemical and phase composition [46]. Coarser particles are enriched in Fe and Si due to the presence of magnetite (Fe_3_O_4_) and fayalite (Fe_2_SiO_4_). In contrast, finer fractions contain higher concentrations of Mg^2+^ and Al^3+^.

The grinding process also generates a significant amount of fine and dust-like particles, which, together with phase composition, further influence leaching intensity. Thus, particle size distribution analysis provides preliminary insights into the mineralogical and chemical characteristics of slag, which is critical for selecting effective processing methods. The particle size distribution of the studied slags prior to grinding is presented in Table 4. Standard deviations are not shown for clarity; however, all measurements were performed in triplicate and met the statistical significance criterion (*p* < 0.05).

**Figure 1:**
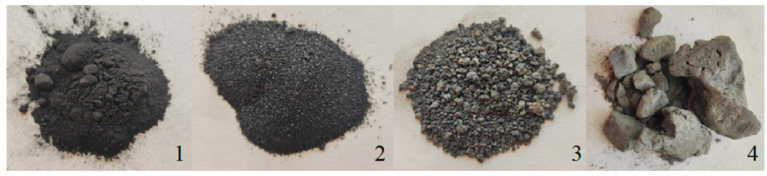
Metallurgical slags: (**1**) slag storage 1 of the former lead plant, (**2**) slag storage 2 of the former lead plant, (**3**) slag storage 3 of the former lead plant, (**4**) slag storage of the Zhezkent MPP.

According to Figure 1 and Table 4, the slags from slag dumps 2 and 3 (former lead smelter) exhibit a finer granulometric profile, with the fraction size below 5 mm accounting for 57.82% and 58.65%, respectively. These samples are characterized by a dense matrix and angular particle morphology, suggesting high mechanical strength. Meanwhile, the slag from dump 1 is the most fragmented, with 4.66% of the material consisting of fractions smaller than 2 mm. Conversely, the slags from the Zhezkent MPP are composed of coarse aggregates (5–15 cm) that readily disintegrate under minimal mechanical stress, indicating low density and high porosity. This material is further distinguished by its friable internal structure and light brown coloration, differing significantly from the other studied samples.

The studied non-ferrous metallurgical slags contain significant quantities of macro- and microelements essential for plant nutrition. According to the literature [5], slags from the Zhezkazgan MPP are characterized by a high content of copper-bearing minerals, including chalcopyrite (CuFeS_2_, 30–50% of the mineral composition), covellite (CuS 5–20%), chalcocite (Cu_2_S, 20%), and bornite (Cu_5_FeS_4_, 4–25%). Additionally, these slags contain galena (PbS), sphalerite (ZnS), and minor amounts of pyrite (FeS_2_), hematite (Fe_2_O_3_), magnetite (Fe_3_O_4_), arsenopyrite (FeAsS), and rutile (TiO_2_), along with inclusions of native gold (Au). Moreover, historical data [7] indicate that slags from the former lead plant contain up to 22.2% SiO_2_, 46% metal oxides and 17.8% C, as well as significant amounts of Zn (9.08%) and Pb (4.22%). It has been noted [12] that lead slags predominantly consist of an approximately 80% glassy CaO–FeO–SiO_2_ matrix. However, during long-term storage, slags may undergo chemical transformation under environmental influences such as solar radiation, precipitation, and temperature fluctuations. Therefore, macroelements (Ca, Mg), microelements (Cu, Zn, Fe, Mn) and potentially toxic elements (As, Cd, Cr, Pb) were analyzed to evaluate the feasibility of chemical leaching for the production of liquid fertilizers. The results of the elemental analysis are summarized in Table 5 [47].

As shown in Table 5, the elemental composition of the slags collected from three dumps of former lead smelter and the Zhezkent Mining and Processing Plant exhibits substantial variation. All samples are characterized by a high Fe content, ranging from 150,900 to 261,000 mg/kg. Elevated concentrations of Ca, Mg, and K were observed in the slag from dump 1, suggesting its potential agronomic value, provided that Pb is either removed or immobilized. However, significant levels of heavy metals were detected in the slags from dump 3 and the Zhezkent plant, including Pb (17,500 and 2200 mg/kg), Cd (37 and 19 mg/kg), and As (79 and 110 mg/kg), which pose a potential environmental risk. Based on the elemental analysis, the sample from slag dump 2 (former lead smelter) was selected for chemical leaching due to its low concentration of potentially toxic elements (PTE). This selection aims to evaluate the feasibility of using the resulting extracts for fertilizer applications.

The subsequent stage of this study focused on the extraction of valuable elements from metallurgical slags. Chemical leaching, employing salts and alkalis as leaching agents, was selected as an energy-efficient and environmentally friendly approach for slag processing. Two reagents were investigated: aqueous 15% potassium sulfate (K_2_SO_4_) and 25% ammonium hydroxide (NH_4_OH). The first reagent, K_2_SO_4_, reacts with metal oxides present in the slag to form KOH, which serves as a potassium source, along with metal sulfates that provide essential trace elements (Reaction 5). The second reagent, NH_4_OH, a widely used agent in hydrometallurgical processes (Reactions 6–9), acts as a source of nutrient nitrogen (N) [48].

In both systems, leaching was conducted in the presence of hydrogen peroxide (H_2_O_2_) as an oxidizing agent. H_2_O_2_ plays a crucial role by promoting iron oxidation and facilitating its efficient removal from the system. Its presence also enhances oxidative conditions, increasing the solubility of metal components and thereby improving overall leaching efficiency [49].

FeO + K_2_SO_4_ + H_2_O_2_ = Fe_2_O_3_ + H_2_SO_4_ + KOH (5)

2CuFeS_2_ + 16NH_3_ + 8.5O_2_ + (n + 2)H_2_O = 2Cu(NH_3_)_4_SO_4_ + 2(NH_4_)_2_SO_4_ + +Fe_2_O_3_·nH_2_O (6)

Cu_2_AsS_4_ +16NH_3_ + 8.75O_2_ + 2.5H_2_O = 3Cu(NH_3_)_4_SO_4_ + (NH_4_)_2_SO_4_ + (NH_4_)_2_HAsSO_4_ (7)

CuS + 4NH_3_ + 2O_2_ = Cu(NH_3_)_4_SO_4_ (8)

Cu_2_S + 6NH_3_ + (NH_4_)_2_SO_4_ + 2.5O_2_ = 2Cu(NH_3_)_4_SO_4_ + H_2_O (9)

The results in Table 6 indicate that leaching with the K_2_SO_4_ solution extracts higher quantities of Ca, Zn, Mn, and Pb compared to the NH_4_OH solution. A primary advantage of ammonia leaching is the selective recovery of Cu, which forms soluble ammine complexes such as [Cu(NH_3_)_4_]^2+^. Consequently, the NH_4_OH solution extracts approximately four times more Cu than the K_2_SO_4_ system. Both extracts exhibit similar concentrations of Mg, Fe, and Cd, while Cr and As remain below the detection limit. The relatively low recovery rates, despite high elemental concentrations in the initial slag, may be attributed to the presence of poorly soluble oxides and silicate phases, such as fayalite and magnetite. The leaching efficiency is likely influenced by the distribution of elements between vitreous and crystalline phases, slag cooling rates (e.g., rapid quenching vs. slow cooling), and the presence of residual sulfur or fluxes. A common challenge in recovering metals from slag via solvent extraction is silica precipitation, which can complicate the process [50]:

2FeO·SiO_2_ + 2H_2_SO_4_ -> 2FeSO_4_ + H_4_SiO_4_ ↓ (10)

Thus, the obtained results demonstrate the potential for selective element extraction using the investigated reagents. The extraction efficiency is governed by the chemical composition of the slag and the nature of the leaching agent.

The resulting metallurgical slag extracts were evaluated as mineral fertilizers to assess their impact on the growth and yield of maize. To establish a baseline for fertility assessment, the agrochemical properties and texture of the experimental soil were analyzed. The soil texture analysis results are presented in Table 7. Values are presented as mean values (*n* = 3).

According to Kachinsky’s classification, the investigated soil belongs to the category of loose sands in terms of mechanical composition [51]. Loose sandy soils are characterized by low strength and weak structural stability, which limits their water-holding capacity and increases susceptibility to erosion [52]. In such soils, nutrients are rapidly leached, thereby reducing crop yields and the overall effectiveness of fertilizers [53].

The results of the agrochemical analysis of the soil prior to planting, used for the pot experiments, are presented in Table 8. The values are expressed as mean ± confidence interval (*n* = 3).

The obtained agrochemical data reveal low concentrations of organic matter, as well as macro- and microelements, which is consistent with the sandy texture of the studied soil. Loose sandy soils have a limited capacity to retain nutrients and organic matter. According to [55], deep loamy and clay-loam soils are most favorable for corn cultivation, providing more stable yields. To improve seed performance in loose sandy soils, corn seeds were subjected to pre-sowing treatment with fertilizer solutions, aimed at enhancing the early availability of macro- and microelements derived from the slag-based extracts.

The extracts were diluted to a concentration at which seed germination was equal to the control (100% germination). These selected dilutions ensured safe exposure of seeds. Germination energy and laboratory germination were determined in triplicate, with 22 seeds per replicate (66 seeds per treatment). The laboratory test showed good germination of corn seeds under all three experimental conditions. Eighteen seeds germinated on day 4, corresponding to a germination energy of 81%, while 22 seeds germinated by day 7, resulting in a final germination of 100%. These results indicate that the fertilizer solutions do not adversely affect seed germination and provide additional nutrients at early stages, which is particularly important for loose sandy soils prone to rapid nutrient leaching.

After 7 days, the germinated seeds were transplanted into soil. A total of 22 plants per treatment variant were used for further observations. Morphometric growth parameters 90 days after planting are presented in Table 9.

Application of an NH_4_OH-based extract positively influenced plant growth parameters, particularly root development. The fresh and dry root biomass in this group (12.45 ± 0.71 g and 11.06 ± 0.84 g, respectively) was higher than that in the control group (8.97 ± 0.75 g and 8.12 ± 0.54 g). Although stem height and length showed only slight increases, improved root system development suggests a potential synergistic effect. This stimulation is likely attributed not only to the early-stage nitrogen availability but also to the presence of micronutrients mobilized from the slag (Table 6, such as Cu and Zn, which are known to support seedling vigor [56]. In contrast, the K_2_SO_4_-based extract primarily promoted cob development and dry biomass accumulation. The presence of Ca, Mg, and Cu in the potassium extract may have further enhanced nutrient uptake efficiency through synergistic ion interactions.

Fertilizer use influences the elemental composition of plant organs. Furthermore, the widespread use of NPK fertilizers contributes to increased yields. However, the use of micronutrient fertilizers is associated with certain difficulties, as plants require low concentrations of micronutrients. Therefore, micronutrients are most often incorporated directly into base fertilizers during their production. The results of elemental analysis of corn plant organs are presented in Table 10.

For elemental analysis, toxicants with low MAC values in plant tissues according to Kabata-Pendias (Table 10) were selected, to assess the potential phytotoxicity of the studied fertilizer.

As shown in Table 11, the concentrations of the studied elements in corn organs do not exceed the established MAC values. When seeds were soaked in an extract containing K_2_SO_4_ with elevated levels of K, Ca, Mg, Cu, Zn, Fe, and Mn, plants accumulated Cu and Pb in the roots, stems, and leaves, while Cr was mainly accumulated in the leaves. Soaking seeds in an extract containing NH_4_OH promoted the accumulation of Zn in the stems of mature plants.

In accordance with the recommendations of Kabata-Pendias and Pendias [22] (Table 11), the translocation coefficient (TC) of elements is determined to assess the degree of accumulation of chemical elements in plant organs (Table 12).

The translocation coefficient (TC) represents the ratio of the average element concentration in aboveground plant organs (stems, leaves, and cobs) to the average concentration in roots.

TC confirmed the general distribution pattern for Cr, Pb, and Cu in plants: roots > stems > leaves. When seeds were treated with K_2_SO_4_, intensive Cr accumulation was observed in the stems relative to the roots. Cr translocation is likely related to the presence of mobile ions in the K_2_SO_4_ extract. The TC of Pb in stems was significantly lower than in leaves, reflecting the limited mobility of Pb. When seeds were treated with NH_4_OH, intensive accumulation was observed for Zn, which is related to the synergistic interaction between Zn and N.

## 3. Materials and Methods

### 3.1. Sampling and Sample Preparation of Metallurgical Slags

Slag samples were collected from three slag dumps of the former Shymkent lead smelter and from one slag dump of the Zhezkent MPP in October 2024 (Figure 1). Spot samples were collected following a systematic sampling route at a depth of 0–10 cm. The total mass of each composite sample was 8–10 kg. After collection, the material was homogenized on a clean surface using a spatula, then air-dried and stored in a dry, ventilated area. The air-dried samples were subjected to particle size distribution and elemental analysis.

### 3.2. Slag Granulometric Analysis

The granulometric composition of the slag was determined using the sieve method with a sieve analyzer (MITR ZDS-200W, Zhengzhou, China). A 200 g sample of the representative slag was placed on the top sieve and sifted through sieves with aperture diameters of >5; 5–2; 2–1; 1–0.75; 0.75–0.5; 0.5–0.25; and <0.25 mm for 10 min. After sifting, each fraction was weighed on an analytical balance with an accuracy of ±0.0001 g. The mass fraction of each fraction was calculated according to Equation (11):

(11) $$\omega =\frac{m}{M}\;\times \;100\%$$

where ω is the mass fraction (%); m is the fraction mass (g); M is the total slag sample mass (g).

### 3.3. Elemental Analysis of Slags

Elemental analysis of solid slags was performed using inductively coupled plasma mass spectrometry (ICP-MS) with an Agilent 7700x mass spectrometer (Agilent Technologies, Santa Clara, CA, USA) and an iCAP 6300 Duo inductively coupled plasma optical emission spectrometer (ICP-OES, Thermo Scientific, Waltham, MA, USA), following the method described in Sabitova et al. [44,57]. Experimental data were processed using statistical methods, including variation, correlation, and regression analyses, implemented in STATISTICA (version 10.0) and Microsoft Excel.

### 3.4. Chemical Leaching of Slags and Elemental Analysis of Leachates

Chemical leaching was performed using a 15% K_2_SO_4_ solution and a 25% NH_4_OH solution following Mikula et al. [35]. To prepare a 15% K_2_SO_4_ solution, 150 g of salt was dissolved in 0.85 L of distilled water. To obtain 10% H_2_O_2_, solution, a 37% H_2_O_2_ solution was diluted with distilled water in a ratio of 1:2.7. A 25% NH_4_OH solution was used without further dilution.

A 50 g slag sample with a particle size of <1.0 mm obtained after preliminary grinding was mixed with 100 mL of leaching solution consisting of the leaching agent and 10% H_2_O_2_ at a volume ratio of 1:1. The solid-to-liquid ratio was maintained at 1:2 (S:L = 1:2). The mixture was placed in 500 mL conical flasks and subjected to leaching. The flasks were placed on a laboratory shaker (Ikeme Lab, Guangzhou, China) for 1 h and subsequently centrifuged using a centrifuge (ELMI SkyLine CM-6M, Riga, Latvia). After separation of the solid residue, the resulting extract was diluted with distilled water at a 1:10 volume ratio and analyzed for elemental composition using an inductively coupled plasma mass spectrometer (ICP-MS, Varian 820, Macquarie Park, NSW, Australia) [44,57]. The results are presented as the mean ± confidence interval calculated for three repetitions (*n* = 3) using Student’s t-distribution at a significance level of *p* < 0.05.

### 3.5. Soil Sampling and Sample Preparation

Soil sampling was carried out in a residential area of Semey, Republic of Kazakhstan. Sampling was performed using the quadrat method over a total area of 16 m^2^. Within each quadrat, soil samples were collected from five points at a depth of 0–20 cm (surface horizon) and combined to obtain a composite sample weighing approximately 100 g. The samples were air–dried, homogenized, and sieved. The mechanical (particle size) composition of the soil was determined by the dry sieving method using a set of laboratory sieves with mesh sizes ranging from 5.0 to 0.25 mm, arranged sequentially from coarse to fine fractions.

Soil agrochemical properties, including hygroscopic moisture content, actual pH, organic matter content, total nitrogen (Kjeldahl method), and elemental composition, were determined using standard physicochemical methods. The 1–2 mm air-dried soil fraction was used for all analyses.

For pH determination, a soil suspension was prepared in distilled water at a soil-to-solution mass ratio of 1:2.5. The suspension was shaken for 60 min using a laboratory shaker (Ikeme Lab, Guangzhou, China) and allowed to settle for 60 min. It was then stirred again for 10 s, after which the pH was measured using a pH meter (INESA ZDJ-4A, Shanghai, China) equipped with a glass electrode.

The organic matter content was determined by the gravimetric loss-on-ignition method. Soil samples were calcined in a muffle furnace (SNOL LSF01, Utena, Lithuania) at 525–550 °C until constant mass was achieved. The organic matter content was calculated according to Equation (12):

(12) $$\omega \;=\;\frac{m_{1}-m_{2}}{m_{1}}\;\times \;100\%$$

where m_1_ is the dry sample mass, g; m_2_ is the sample mass after ignition, g.

For elemental analysis, acid digestion of dry soil was performed. A 2.00 g sample of soil was placed in a round-bottomed flask, 20 mL of 3% HNO_3_ solution was added, and the mixture was shaken on a reciprocating shaker (Ikeme Lab, Guangzhou, China) for 60 min. After settling, the resulting soil suspension was filtered through blue ribbon filter paper. The filtrate was then diluted with distilled water at a 1:50 volume ratio and analyzed using ICP-MS (VARIAN 820, Macquarie Park, NSW, Australia). Equation (13) was used to calculate the concentration of chemical elements in the sample:

(13) $$C=\frac{a\times 50}{2\times 1000}$$

where C is the element concentration, mg/g; a is the element concentration value displayed by the instrument, mg/L; 50 is the extract volume, mL; 2 is the soil sample weight, g; 1000 is the conversion factor.

### 3.6. Determining the Laboratory Germination of Zea mays L. Seeds

To determine the bioactivity of metallurgical waste extracts, they were diluted with distilled water. Filter paper in plastic containers was then sprayed with 10.5 mL of the solution using a spray bottle. The following treatments were used: 1—control (H_2_O), 2—1:200 solution (NH_4_OH), 3—1:10 solution (K_2_SO_4_). The elemental composition (mg/L) of the extracts after dilution is shown in Table 13.

Corn seeds (22 seeds per 17 × 26 cm container) were placed between layers of moistened filter paper rolled into rolls. The hermetically sealed containers with seeds were germinated in a light-tight growbox at a temperature of 20–25 °C. The containers were ventilated once daily for 30 min and moistened as needed with distilled water [45]. Germination energy was determined on day 4, and germination rate on day 7, according to Equations (14) and (15):

(14) $$E=\frac{number\;of\;germination\;seeds}{total\;number\;of\;seeds\;in\;the\;sample}\;\times \;100\%$$

(15) $$G=\frac{viable\;seeds}{total\;number\;of\;seeds\;in\;the\;sample}\;\times \;100\%$$

where E is the seed germination energy, %; G is the laboratory germination rate, %.

### 3.7. Elemental Analysis of Zea mays L. Plant Organs

To study the subsequent development of the plants, small-plot field pot experiments were conducted. Plant seedlings obtained from the laboratory germination experiment were transplanted into open field conditions and grown for 90 days, with regular irrigation and mechanical weed removal. Field experiment variants: (1) seeds treated with an NH_4_OH-based extract; (2) seeds treated with a K_2_SO_4_-based extract; (3) seeds without extract treatment (control). At the end of the experiment, plant height, stem diameter, number of leaves and cobs, and fresh and dry biomass of the aboveground parts and roots were determined.

Samples of leaves, stems, roots, and cobs were washed and dried in a drying oven (SNOL 58/350, Utena, Lithuania) at 105 °C to constant weight, after which they were ground to a homogeneous state in a coffee grinder. For dry ashing, a 5 g portion of the dried sample was transferred to porcelain crucibles and calcined in a muffle furnace (SNOL LSF01, Utena, Lithuania) at 450–500 °C for 5–8 h. Ash extraction was performed in concentrated HNO_3_ (65%), at a solid phase (g) to solution (mL) ratio of 1:10. The reaction was carried out in an ADS Multi Acid Digestion System (Spectromart, Moscow, Russia) at 120 °C for 2 h. The extraction solutions were passed through blue ribbon filters and their volume was adjusted to 50 mL with distilled water. The concentrations of Ca, Mg, Cu, Zn, Fe, Mn, Cr, Cd, Pb, and As in the resulting extracts were determined using an ICP-MS (VARIAN, Macquarie Park, NSW, Australia) [44]. Statistical analysis of the experimental data was carried out using Student’s t-test to determine significant differences between the variants and the control group. All measurements were performed in three replicates.

The translocation coefficient (TC), defined as the ability of PTE to be transferred from the roots to the aboveground parts of agricultural crops, was calculated using Equation (16):

(16) $$TC\;=\;\frac{C_{1}}{C_{2}}$$

where c_1_ is the average concentration of PTE in the aboveground parts of the plant, mg/kg; c_2_ is the average concentration of PTE in the roots, mg/kg.

## 4. Conclusions

Slags are anthropogenic waste produced by the metallurgical industry, and their efficient processing is an important task for environmental protection.

Elemental analysis revealed that slag from dump 2 of the former Shymkent lead smelter had the lowest PTE content. Chemical leachates obtained using potassium sulfate exhibited more than three times higher concentrations of Ca, Zn, and Mn compared to the NH_4_OH-based system. In contrast, NH4OH-based leachates demonstrated more than four times higher Cu extraction efficiency.

The bioactivity of the obtained extracts was assessed using seed germination and plant growth assays of *Zea mays* L. A 100% laboratory germination rate was established after pre-sowing treatment by soaking in diluted extracts of 1:10 K_2_SO_4_:H_2_O and 1:200 NH_4_OH:H_2_O. Seed treatment with the ammonia extract had the most pronounced effect on the root system, stems, and cobs of the mature plants. Elemental analysis of plant tissues revealed the accumulation of Cu after seed treatment with the K_2_SO_4_ extract and Zn after treatment with the NH_4_OH extract, with no detectable accumulation of toxic metals. The experiment demonstrates the potential of using metallurgical slag extracts in the production of liquid mineral fertilizers. This approach enables the valorization of metallurgical waste into environmentally safe fertilizers with confirmed agronomic efficiency.

## Figures and Tables

**Table 1:** Volumes of metallurgical slags from non-ferrous and ferrous metallurgy.

Produced Metal	Volume of Generated Slag, t	Reference
Iron (steel)	0.12	[10]
Iron (cast iron)	0.22–0.37	[11]
Lead	0.6–0.7	[12]
Aluminum	0.2–0.5	[13]
Ferrochrome	1.1–3.5	[14]
Zinc	>2	[15]
Copper	2.2	[9,16,17]
Magnesium	>6	[18]
Nickel	6–16	[19]

**Table 2:** Comparative content of potentially toxic chemical elements in metallurgical slags, mg/kg.

Element	Ferrous Metallurgy Slag	Non-Ferrous Metallurgy Slag	Maximum Allowable Concentration (MAC) in Soil [22,23]
Cd	≤128	≤14,000	5
Pb	0.2–126	≤319,190	32
As	≤244	75,865	2
Cr	0.1–32,700	≤7510	6
Co	0.03–210	0.97–24,104	5
Zn	0.15–11,000	13–379,694	23
Cu	0.13–540	5–353,580	3
Fe	0.02–61.8	0.67–62.0	2000–15,000

**Table 3:** Studies on the use of metallurgical wastes to improve crop yields and soil fertility.

Metallurgical Slag	Effects of Application in Agriculture
Steel slag	Application at 20 g/kg soil enhanced the growth parameters of *Capsicum annuum* L. by twofold or more, with sulfur (S) content in fruits exceeding the positive control fourfold. Concentrations of other macro- and microelements (N, P, K, Ca, Mg, Zn, Fe, Si) in fruits remained comparable to those in the positive control [28].
Dried sludge from wet gas cleaning in a blast furnace shop and converter slag	Application increased the field yield of *Avena sativa* L. by over 30%, with plant height increasing by an average of 18% [29].
Linz-Donawitz converter slag	The addition increased the content of organic carbon in the soil by 14% and easily mineralized carbon by 42%, while the carbon content of microbial biomass and available phosphorus increased by 30% and 33%, respectively. In addition, the exchangeable Ca^2+^ increased by 47%, and exchangeable Mg^2+^ by 65%. It also enhanced the photosynthetic rate in *Oryza sativa* L. by 21.1% and 18% in Japonica and Indica varieties, respectively. In addition, straw nutrient contents increased, with N (20.1–22.2%), P (17–18.4%), and Si (29.9–30.5%) in both varieties. These improvements led to increased productivity, as grain yield, straw biomass, and root biomass increased by 15.2% and 13.6%, 19.9% and 22%, and 17.2% and 19.4% in Japonica and Indica varieties, respectively [30].
Steel slag	Simultaneous application of steel slag (2% by weight) and vermicompost (4% by weight) increased soil electrical conductivity by 34% and microbial growth rate by 119%, while reducing Cu bioavailability in contaminated soils by 72%. Biomass of *Lolium Perenne* L. under this treatment exceeded that of the vermicompost-only variant by 15% [31].
Electric arc furnace (EAF) slag	The introduction of low-slag EAF additives (EAF slag + NPK) into the soil improved gas exchange parameters, with the net photosynthetic rate being 30% higher under than with NPK alone. It enhanced nitrate reductase activity in the bean plant *Phaseolus vulgaris* L. [32].
Steel slag	Application of water-cooled slag or steel slag fertilizer increased the yield of table grapes *Vitis vinifera* L. by 13.5% compared to the control after two years of testing [33].
Blast furnace gas cleaning sludge	The introduction of highly dispersed blast furnace gas cleaning sludge (0.5–2 t/ha) stimulated the photosynthetic activity of *Brassica napus* L. plants, increasing the average root and stem lengths by 50% and 15%, respectively. Maximum seed germination was also observed, exceeding control values by 7% [34].
Lead slag	The introduction of a 20% dose of lead slag extract increased the biomass of *Cucumis sativus* L. seedlings by 11% compared to the control [35].
Magnesium slag	Application of magnesium slag fertilizer increased lodging resistance, enhanced late-stage growth, and shortened the growing season of agricultural crops, including *Zea diploperenni* L., *Raphanus sativus* L. [36].

**Table 4:** Results of particle size analysis of slags.

Fraction Size, mm	Content, %			
	Slag from Former Lead Plant, Storage Facility 1	Slag from Former Lead Plant, Storage Facility 2	Slag from Former Lead Plant, Storage Facility 3	Slag from Zhezkent MPP
>5	83.32	42.18	41.35	89.30
5–2	11.31	22.31	23.95	0.27
2–1	1.66	18.06	16.72	0.60
1–0.75	1.63	9.93	10.05	0.25
0.75–0.5	0.68	4.24	4.35	0.75
0.5–0.25	0.61	2.05	2.15	4.07
<0.25	0.80	1.25	1.45	4.77
Total	100.01	100.02	100.02	100.01

**Table 5:** Elemental analysis of metallurgical slags before chemical leaching, mg/kg.

Element	Slag from Former Lead Plant, Storage Facility 1	Slag from Former Lead Plant, Storage Facility 2	Slag from Former Lead Plant, Storage Facility 3	Slag from Zhezkent MPP
K	10,600 ± 1400	7500 ± 980	4000 ± 540	6100 ± 830
Ca	93,800 ± 12,200	92,800 ± 12,000	40,500 ± 5400	19,400 ± 2500
Mg	17,800 ± 2300	15,400 ± 2000	13,900 ± 1900	18,700 ± 2500
Cu	5100 ± 670	7200 ± 940	3700 ± 500	1800 ± 250
Zn	<d.l.	8600 ± 1200	87,400 ± 15,600	4400 ± 650
Fe	256,000 ± 33,000	259,000 ± 34,000	261,000 ± 34,700	150,900 ± 19,800
Mn	4400 ± 580	4900 ± 640	3800 ± 510	400 ± 50
Na	10,700 ± 1400	9200 ± 1200	5100 ± 670	2600 ± 360
Pb	860 ± 110	460 ± 60	17,500 ± 2300	2200 ± 290
Cr	<d.l.	<d.l.	65 ± 11	10 ± 1
Cd	<d.l.	1.4 ± 0.2	37 ± 6	19 ± 3
As	<d.l.	8 ± 1	110 ± 19	79 ± 12

Note: “< d.l. “—below the detection limit.

**Table 6:** Elemental analysis of metallurgical slag extracts, mg/L.

Element	K_2_SO_4_	NH_4_OH
Ca	27.605 ± 0.199	9.398 ± 0.084
Mg	5.959 ± 0.064	6.088 ± 0.046
Cu	91.365 ± 4.327	423.751 ± 5.750
Zn	86.649 ± 1.094	34.352 ± 0.882
Fe	5.567 ± 0.025	5.028 ± 0.013
Mn	22.652 ± 0.163	0.185 ± 0.001
Pb	3.343 ± 0.108	<d.l.
Cr	<d.l.	<d.l.
Cd	0.393 ± 0.050	0.269 ± 0.001
As	<d.l.	<d.l.

**Table 7:** Texture of the surface soil layer.

Fraction Size, mm	>5	5–2	2–1	1–0.75	0.75–0.5	0.5–0.25	<0.25
Mass fraction, %	12.03	9.29	9.24	14.30	24.62	22.97	7.56

**Table 8:** Chemical characteristics of the soil.

Parameter	Measured Value	Optimum Value [54]
pH in water	8.5	6–7
Organic matter, %	1.2573 ± 0.5157	>5
N, g/kg	11.9 ± 0.03	30–40
K, g/kg	0.0386 ± 0.00015	20–30
P, g/kg	0.2286 ± 0.0086	3–5
Ca, g/kg	0.4853 ± 0.0051	2.5–8
Mg, g/kg	0.0531 ± 0.0001	1.5–6
Cu, mg/kg	0.8 ± 0.05	5–25
Zn, mg/kg	8.4 ± 0.09	20–70
Fe, mg/kg	396.5 ± 7.5	30–250
Mn, mg/kg	3.0 ± 0.1	20–150
Pb, mg/kg	2.3 ± 0.027	-
Cr, mg/kg	12.1 ± 0.01	-
Cd, mg/kg	<d.l.	-
As, mg/kg	<d.l.	-

Note: “-“—no data available.

**Table 9:** Biometric growth parameters of *Zea mays* L. plants.

Biometric Parameter	Seed Treated with K_2_SO_4_ –Based Extract	Seed Treated with NH_4_OH—Based Extract	Control (No Fertilizer Extract Treatment)
Plant height, cm	158.5 ± 10.14	167.5 ± 7.31	165 ± 9.72
Stem length, cm	149.5 ± 15.53	155.5 ± 13.82	154.7 ± 14.35
Root length, cm	25 ± 1.68	22 ± 1.43	24 ± 1.54
Stem diameter, cm	1.56 ± 0.02	1.62 ± 0.01	1.59 ± 0.02
Number of leaves, pcs	13 ± 1	9 ± 1	13 ± 1
Number of cobs, pcs	1	1	1
Cob diameter, cm	4.9 ± 0.15	5.1 ± 0.09	4.9 ± 0.16
Cob length, cm	29 ± 0.71	28 ± 0.49	28 ± 0.65
Fresh plant biomass, g	379.39 ± 29.61	338.93 ± 27.15	381.08 ± 31.67
Dry aboveground biomass, g	115.53 ± 14.67	73.61 ± 8.96	100.36 ± 8.61
Fresh root biomass, g	10.89 ± 0.83	12.45 ± 0.71	8.97 ± 0.75
Dry root biomass, g	10.85 ± 0.21	11.06 ± 0.84	8.12 ± 0.54

**Table 10:** PTE concentrations in *Zea mays* L. plant organs (dry weight, mg/kg).

Plant Organs	Solution	Cr	Pb	Cu	Zn
Roots	K_2_SO_4_	1.515 ± 0.017	6.554 ± 0.387	12.490 ± 0.470	53.146 ± 0.852
	NH_4_OH	0.9617 ± 0.017	6.847 ± 0.501	6.090 ± 0.522	45.348 ± 2.909
	H_2_O	3.029 ± 0.149	10.382 ± 0.350	9.262 ± 0.172	70.218 ± 2.716
Stems	K_2_SO_4_	<d.l.	6.520 ± 0.710	10.328 ± 1.80	41.971 ± 1.619
	NH_4_OH	<d.l.	<d.l.	1.423 ± 0.132	80.627 ± 2.599
	H_2_O	<d.l.	<d.l.	0.446 ± 0.042	104.281 ± 4.978
Leaves	K_2_SO_4_	2.319 ± 0.054	<d.l.	4.341 ± 0.067	44.324 ± 1.116
	NH_4_OH	<d.l.	3.179 ± 0.595	3.078 ± 0.328	53.979 ± 2.827
	H_2_O	<d.l.	3.359 ± 0.053	2.491 ± 0.126	34.112 ± 1.190
Cobs	K_2_SO_4_	<d.l.	<d.l.	1.220 ± 0.221	45.438 ± 0.857
	NH_4_OH	<d.l.	<d.l.	2.739 ± 0.239	55.661 ± 2.233
	H_2_O	<d.l.	<d.l.	3.506 ± 0.199	46.382 ± 5.331
MAC in plants, mg/kg [22]		5–30	30–300	5–30	100–400

**Table 11:** The degree of accumulation of pollutants depending on the TC.

TC Value	Degree of Uptake
TC < 0.01	no uptake
0.01 ≤ TC ≤ 0.1	weak uptake
0.1 ≤ TC ≤ 1.0	moderate uptake
1.0 ≤ TC	intensive uptake

**Table 12:** Translocation coefficients of *Zea mays* L. plant.

Element		Cr	Pb	Cu	Zn
stems/roots	K_2_SO_4_	1.531 *	0.995	0.827	0.789
	NH_4_OH	*-*	*-*	0.233	1.778 *
	H_2_O	*-*	*-*	0.048 **	1.485 *
leaves/roots	K_2_SO_4_	*-*	0.485	0.347	0.834
	NH_4_OH	*-*	0.464	0.505	1.190 *
	H_2_O	*-*	0.324	0.269	0.486
cobs/roots	K_2_SO_4_	*-*	*-*	0.097 **	0.855
	NH_4_OH	*-*	*-*	0.449	1.227 *
	H_2_O	*-*	*-*	0.378	0.661

Note: *—intense accumulation. **—weak accumulation.

**Table 13:** Composition of metallurgical waste extracts.

Element	Ca	Mg	Cu	Zn	Fe	Mn	Pb	Cr	Cd	As
K_2_SO_4_	2.509	0.542	8.306	7.877	0.506	2.059	0.304	<d.l.	0.0357	<d.l.
NH_4_OH	0.047	0.030	2.108	0.171	0.025	0.0009	<d.l.	<d.l.	0.001	<d.l.

## Data Availability

The original contributions presented in the study are included in the article, further inquiries can be directed to the corresponding author.

## Abbreviations

The following abbreviations are used in this manuscript:

MPP	Mining and processing plant
ICP-MS	Inductively coupled plasma mass spectrometer
MAC	Maximum allowable concentration
TC	Translocation coefficient

## References

[1] Spooren J., Binnemans K., Björkmalm J., Breemersch K., Dams Y., Folens K., González-Moya M., Horckmans L., Komnitsas K., Kurylak W. (2020). Near-zero-waste processing of low-grade, complex primary ores and secondary raw materials in Europe: Technology development trends. Resour. Conserv. Recycl..

[2] Jin Z., Liu T., Yang Y., Jackson D. (2014). Leaching of cadmium, chromium, copper, lead, and zinc from two slag dumps with differert environmental exposure periods under dynamic acidic condition. Ecotoxicol. Environ. Saf..

[3] Piatak N.M., Parsons M.B., Seal R.R. (2015). Characteristics and environmental aspects of slag: A review. Appl. Geochem..

[4] Duczmal-Czernikiewicz A., Baibatsha A., Bekbotayeva A., Omarova G., Baisalova A. (2021). Ore minerals and metal distribution in tailings of sediment-hosted stratiform copper deposits from Poland and Kazakhstan. Minerals.

[5] TransformNation (2024). Chapter 2: Analysis of the Current Situation in Kazakhstan. TransforNation. https://transfornation.kz/tpost/oczcbs0jb1-glava-2-analiz-tekuschei-situatsii-v-kaz.

[6] Abilda Z., Daurov D., Daurova A., Zhapar K., Sapakhova Z., Zhambakin K., Shamekova M. (2023). Construction of a geoecological map of dust particles transfer from the surface of the Shymkent lead factory dump. Eurasian J. Ecol..

[7] Zeng J., Luo X., Cheng Y., Ke W., Hartley W., Li C., Jiang J., Zhu F., Xue S. (2022). Spatial distribution of toxic metal (loid)s at an abandoned zinc smelting site, Southern China. J. Hazard. Mater..

[8] Liu X., Zhang C., Yu H., Qian G., Zheng X., Zhou H., Huang L., Zhang F., Zhong Y. (2024). Research on the properties of steel slag with different preparation processes. Materials.

[9] Gabasiane T.S., Danha G., Mamvura T.A., Mashifana T., Dzinomwa G. (2021). Environmental and socioeconomic impact of copper slag—A review. Crystals.

[10] Du C., Gao X., Kitamura S. (2019). Measures to Decrease and Utilize Steelmaking Slag. J. Sustain. Metall..

[11] Talodhikar V.P. (2016). Study of iron and steel slag as a product with respect to physical-chemical properties. IJAET.

[12] Pan D., Li L., Tian X., Wu Y., Cheng N., Yu H. (2019). A review on lead slag generation, characteristics and utilization. Resour. Conserv. Recycl..

[13] Tsakiridis P.E. (2012). Aluminium salt slag characterization and utilization—A review. J. Hazard. Mater..

[14] Sariev O., Kelamanov B., Dossekenov M., Davletova A., Kuatbay Y., Zhuniskaliev T., Abdirashit A., Gasik M. (2024). Environmental characterization of ferrochromium production waste (refined slag) and its carbonization product. Helion.

[15] SMM (2020). Lead and Zinc Summit: Hematite Process–the Best Choice for the Reduction of Solid Waste from Zinc Smelting and the New Technology of Resource Utilization. Metal News (SMM).

[16] Madheswaran C.K., Ambily P.S., Dattatreya J.K., Rajamane N.P. (2014). Studies on use of copper slag as replacement material for river sand in building constructions. J. Inst. Eng. (India) Ser. A.

[17] Gorai B., Jana R.K. (2003). Characteristics and utilisation of copper slag—A review. Resour. Conserv. Recycl..

[18] Lu P., Zhao Y., Zhang N., Wang Y., Zhang J., Zhang Y., Liu X. (2024). Structural characteristics and cementitious behavior of magnesium slag in comparison with granulated blast furnace slag. Materials.

[19] Zhang G., Wang N., Chen M., Cheng Y. (2020). Recycling nickel Environmental slag by aluminum dross: Iron–extraction and secondary slag stabilization. ISIJ Int..

[20] Nowińska K., Adamczyk Z. (2023). Zinc and lead metallurgical slags as a potential source of metal recovery: A Review. Materials.

[21] De Andrade Lima L.R.P., Bernardez L.Z. (2011). Characterization of the lead smelter slag in Santo Amaro, Bahia, Brazil. J. Hazard. Mater..

[22] Kabata-Pendias A. (2011). Trace Elements in Soil and Plants.

[23] Ministry of Justice of the Republic of Kazakhstan Adilet: Information and Legal System of Regulatory Legal Acts of the Republic of Kazakhstan. https://adilet.zan.kz/rus/docs/V2100022595.

[24] Sabitova A., Ualikhanov A., Klivenko A., Kabysheva Z., Aitkaliyeva G., Kassymova Z. (2025). Microbiological extraction of copper and zinc from metallurgical waste. Eng. Sci..

[25] Bosecker K. (1997). Bioleaching: Metal solubilization by microorganisms. FEMS Microbiol. Rev..

[26] Onisei S., Pontikes Y., Van Gerven T., Angelopoulos G.N., Velea T., Predica V., Moldovan P. (2012). Synthesis of inorganic polymers using fly ash and primary lead slag. J. Hazard. Mater..

[27] Chowdhury S. (2023). Recycled smelter slags for In situ and ex situ water and wastewater treatment—current knowledge and opportunities. Processes.

[28] Ouala O., Essadki Y., Khalisse H., Chagiri H., Meddich A., El Khalloufi F., Oudra B. (2024). Evaluation of slag fertilizer potential in *Capsicum annuum* L. cultivation and production. J. Agric. Environ. Int. Dev. (JAEID).

[29] Zakharova O., Baranchikov P., Chebotaryova S., Grigoriev G., Strekalova N., Grodetskaya T., Burmistrov I., Volokhov S., Kuznetsov D., Gusev A. (2024). Metallurgical waste for sustainable agriculture: Converter slag and blast-furnace sludge increase oat yield in acidic soils. Agronomy.

[30] Das S., Gwon H.S., Khan M.I., Jeong T.S., Kim P.J. (2020). Steel slag amendment impacts on soil microbial communities and activities of rice (*Oryza sativa* L.). Sci. Rep..

[31] Wang X., Xue J., He M., Qi H., Wang S. (2024). The effects of vermicompost and steel slag amendments on the physicochemical properties and bacterial community structure of acidic soil containing copper sulfide mines. Appl. Sci..

[32] Radić S., Sandev D., Maldini K., Vujčić Bok V., Lepeduš H., Domijan A.-M. (2022). Recycling electric arc furnace slag into fertilizer: Effects of “Waste Product” on growth and physiology of the common bean (*Phaseolus vulgaris* L.). Agronomy.

[33] Zhang M., Liang Y., Chu G. (2017). Applying silicate fertilizer increases both yield and quality of table grape (*Vitis vinifera* L.) grown on calcareous grey desert soil. Sci. Hortic..

[34] Zakharova O., Baranchikov P., Grodetskaya T., Kuznecov D., Gusv A. (2022). Highly dispersed blast-furnace sludge as a new micronutrients fertilizer: Promising result on rapeseed. Agronomy.

[35] Mikula K., Skrzypczak D., Izydorczyk G., Baśladyńska S., Szustakiewicz K., Gorazda K., Moustakas K., Chojnacka K., Witek-Krowiak A. (2022). From hazardous waste to fertilizer: Recovery of high-value metals from smelter slags. Chemosphere.

[36] Xia D.H., Ren L., Chen L.Z. (2011). Study of Ca-Mg-S-Si fertilizer produced by magnesium slag. Adv. Mater. Res..

[37] Sagwal A., Wadhwa P., Shubham, Kaushal S. (2023). Essentiality of micronutrients in soil: A Review. Int. J. Plant Soil Sci..

[38] Assunção A.G.L., Cakmak I., Clemens S., González-Guerrero M., Nawrocki A., Thomine S. (2022). Micronutrient homeostasis in plants for more sustainable agriculture and healthier human nutrition. J. Exp. Bot..

[39] Hänsch R., Mendel R. (2009). Physiological functions of mineral micronutrients (Cu, Zn, Mn, Fe, Ni, Mo, B, Cl). Curr. Opin. Plant Biol..

[40] Gautam S., Paudel M.R., Devkova A. (2025). Heavy metal pollution and phytoremediation-a review. Nepal J. Bot..

[41] Retamal-Salgado J., Hirzel J., Walter I., Matus I. (2017). Bioabsorption and bioaccumulation of cadmium in the straw and grain of maize (*Zea mays* L.) in growing soils contaminated with cadmium in different environment. Int. J. Environ. Res. Public Health.

[42] Elik Ü., Gül Z. (2025). Accumulation potential of Lead and cadmium metals in maize (*Zea mays* L.) and effects on physiological-morphological characteristics. Life.

[43] Figlioli F., Sorrentino M.C., Memoli V., Arena C., Maisto G., Giordano S., Capozzi F., Spagnuolo V. (2019). Overall plant responses to Cd and Pb metal stress in maize: Growth pattern, ultrastructure, and photosynthetic activity. Environ. Sci. Pollut. Res..

[44] Sabitova A., Mukhamediyarov N., Mussabayeva B., Rakhadilov B., Aitkazin N., Bayakhmetova B., Sharipkhan Z., Gaisina B. (2025). The effect of the granulometric composition of slags on the efficiency of non-ferrous metal extraction. Processes.

[45] Khaeim H., Kende Z., Jolánkai M., Kovács G.P., Gyuricza C., Tarnawa Á. (2022). Impact of temperature and water on seed germination and seedling growth of maize (*Zea mays* L.). Agronomy.

[46] Riboldi A., Cornacchia G., Gelfi M., Borgese L., Zacco A., Bontempi E., Boniardi M.V., Casaroli A., Depero L.E. (2020). Grain size effect in elution test of electric arc furnace slag. Appl. Sci..

[47] Herbelin M., Bascou J., Lavastre V., Guillaume D., Benbakkar M., Peuble S., Baron J.-P. (2020). Steel slag characterisation—Benefit of coupling chemical, mineralogical and magnetic techniques. Minerals.

[48] Sabitova A., Kassymova Z., Mukiyanova R., Bayahmetova B.B., Nurgaliev N. (2025). Investigation of the effectiveness of metallurgical slags in fertilizer production. Acad. Sci. J. Chem..

[49] Mikula K., Izydorczyk G., Skrzypczak D., Moustakas K., Witek-Krowiak A., Chojnacka K. (2021). Value-added strategies for the sustainable handling, disposal, or value-added use of copper smelter and refinery wastes. J. Hazard. Mater..

[50] Banza A., Gock E., Kongolo K. (2002). Base metals recovery from copper smelter slag by oxidising leaching and solvent extraction. J. Hazard. Mater..

[51] Kachinsky K.N. (1965). Soil Physics.

[52] Wang Y., Li Y., Li Y. (2020). Land engineering consolidates degraded sandy land for agricultural development in the largest sandy Land of China. Land.

[53] Serrapica F., Di Mola I., Cozzolino E., Ottaiano L., Sarubbi F., Pezzullo G., Di Francia A., Mori M., Masucci F. (2025). Sustainable Maize Forage Production: Effect of organic amendments combined with microbial biofertilizers across different soil textures. Sustainability.

[54] Amissah S., Ankomah G., Agyei B.K., Lee R.D., Harris G.H., Cabrera M., Franklin D.H., Diaz-Perez J.C., Habteselassie M.Y., Sintim H.Y. (2023). Nutrient sufficiency ranges for corn at the early growth stage: Implications for nutrient management. Plants.

[55] Oberle S., Keeney D. (1990). Soil type, precipitation, and fertilizer N effects on corn yields. J. Prod. Agric..

[56] Yin M., Li Y., Hu Q., Yu X., Huang M., Zhao J., Dong S., Yuan X., Wen Y. (2023). Potassium increases nitrogen and potassium utilization efficiency and yield in foxtail millet. Agronomy.

[57] Mukhamediyarov N., Sabitova A., Nurgaliev N., Ualihanov A., Aitkazin N. (2025). Current state of metallurgical production waste in southern Kazakhstan and prospects for its processing. Acad. Sci. J. Chem..

